# Granulomatous mastitis – Case report

**DOI:** 10.1016/j.ijscr.2025.111222

**Published:** 2025-03-27

**Authors:** Catarina Gil Ribeiro, Alice Pimentel, Rita Lages, Ana Isabel Ferreira, João Barros da Silva

**Affiliations:** Departamento de Cirurgia de ULS Tâmega e Sousa, Penafiel, Portugal

**Keywords:** Breast, Case report, Granulomatous mastitis, Erythema nodosum

## Abstract

**Introduction and importance:**

Idiopathic granulomatous mastitis (IGM) is a rare, benign, and chronic inflammatory breast disease with unclear etiology and no standardized treatment. It often presents as a painful breast mass that mimics breast cancer, necessitating thorough diagnostic evaluation.

**Case presentation:**

This report discusses a case of a 39-year-old pregnant woman with IGM associated with erythema nodosum, a poorly understood co-occurrence. Initial treatment included antibiotics and surgical drainage for an abscess. Despite multiple interventions, including corticosteroids and methotrexate, the disease persisted with recurrent episodes.

**Clinical discussion:**

This case underscores the complexities of managing IGM, highlighting its association with systemic manifestations such as erythema nodosum. Current therapeutic approaches range from conservative management to immunosuppressive therapy and surgery, with outcomes varying widely.

**Conclusion:**

Further studies are needed to elucidate the pathophysiology and establish optimal treatment strategies for IGM, particularly in the context of systemic involvement.

## Highlights

• Idiopathic granulomatous mastitis mimics breast cancer, requiring thorough diagnosis.
• Often coexists with erythema nodosum, but the link remains poorly understood.
• Management includes antibiotics, corticosteroids, and surgical interventions.
• Recurrence and complications are common, necessitating prolonged follow-up.
• Further research is needed to clarify pathophysiology and optimize treatment.

## 1 Introduction

Idiopatic granulomatous mastitis, also known as chronic granulomatous mastitis or granulomatous lobular mastitis, was first described by *Kessler* and *Wolloch* in 1972 [1]. This is a relatively rare, benign, chronic, and recurrent inflammatory breast disease, accounting for 1.6 % of all breast diseases. Histologic findings include noncaseating granulomas centered on lobules [2]. Its etiology remains unclear, and there is a lack of optimal treatment pathways [3,4].

Various infectious etiologies, autoimmune and connective tissue diseases, trauma, diabetes among other have been implicated in secondary cases of granulomatous mastitis [5].

The condition typically affects non-white (Middle Eastern or Hispanic) women, of reproductive age [2,6]. Patients most often present with a painful mass and may exhibit symptoms similar to those of an abscess or infection. Granulomatous mastitis can mimic breast cancer; therefore, an appropriate diagnostic workup with imaging and core needle biopsy is necessary. While knowledge about the pathophysiology of this condition is limited, immune-mediated mechanisms are believed to play a role. The coexistence of granulomatous mastitis and erythema nodosum has been described, although this association is still poorly understood [2,4,6].

Management remains controversial and is typically multimodal. Medical management may include the use of steroids, methotrexate, and/or antibiotics. Surgical excision may be warranted for complicated or refractory cases.

## 2 Case presentation

**Fig. 1:**
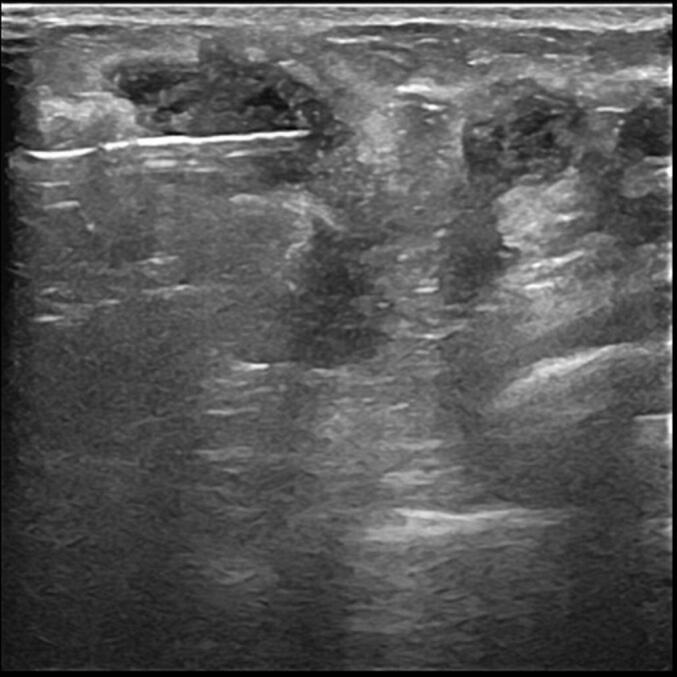
The ultrasound findings showed a hypoechoic formation with well defined and lobulated contours, wich was biopsied.

In this case report, prepared in adherence to the SCARE 2023 guidelines for surgical case reporting [7], we present the case of a 39-year-old Tunisian pregnant woman with a 16 weeks twin pregnancy who consulted our service with a 1-month history of pain and induration of the left breast. A breast ultrasound revealed a 43 mm heterogeneous mass located at the junction of the left external quadrants, with irregular and ill-defined margins. The patient underwent an ultrasound-guided biopsy of the lesion (Fig. 1) that revealed marked inflammatory infiltrate consisting predominantly of neutrophils, some lymphocytes and plasma cells, located in the mammary lobules with permeation and destruction of the acini/breast ducts.

**Fig. 2:**
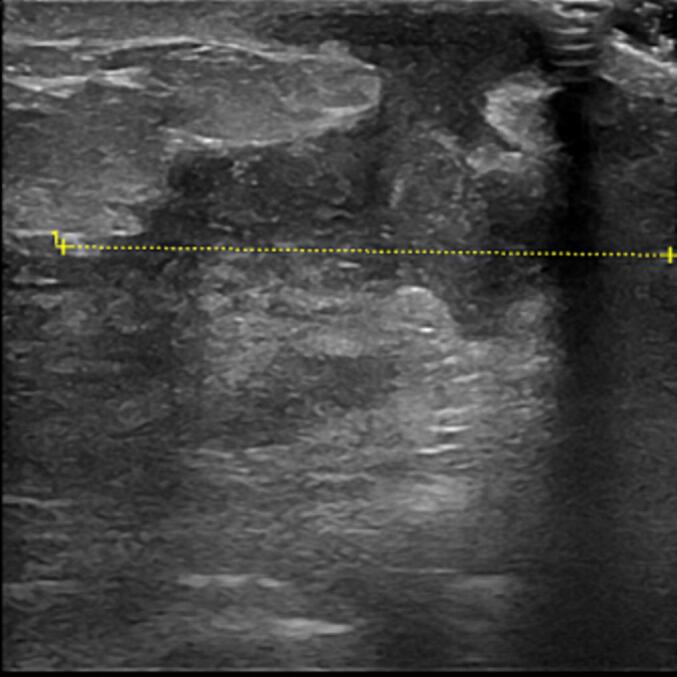
The ultrasound findings showed multiple conflent hypoechoic loculations with ill-defined contours, some of which communicate with the skin, these findings are consistent with mastitis associated with multiple abscessed loculations.

Two weeks later, the patient presented to the emergency department with worsening local inflammatory signs and increased pain in the left breast region. A breast ultrasound was performed, revealing findings consistent with mastitis (Fig. 2). She also exhibited cutaneous nodules with erythema and fluctuance, painful on palpation, located in the elbow region and the anterior tibial surface. Upon this finding the patient reported that she had noticed the lesions for approximately 4 days. A clinical diagnosis of erythema nodosum was made. The patient was evaluated by the internal medicine physician, who recommended a comprehensive blood analysis and a chest X-ray, which the patient refused. She opted to leave the emergency department and was prescribed a 2-week course of antibiotic therapy with amoxicillin-clavulanic acid. After this time the patient was evaluated at the breast clinic, where she presented with a new worsening of local inflammatory signs, now associated with an abscess.

Surgical drainage of the breast abscess was performed, and the patient was hospitalized, receiving antibiotic therapy with piperacillin-tazobactam for 10 days. During the patient's hospitalization, the skin lesions showed gradual improvement, nearly returning to their baseline condition by the time of discharge. A subsequent ultrasound-guided biopsy showed “mammary tissue exhibits a pronounced macrophagic inflammatory infiltrate with scattered multinucleated giant cells and the formation of epithelioid granulomas, predominantly located within the lobules. This process is associated with numerous neutrophilic polymorphonuclear cells, either dispersed or infiltrating mammary ducts and acini. Tests for fungal organisms and staining with periodic acid-Schiff (PAS) and Ziehl-Neelsen were negative for pathogens.”

These clinical findings raised the suspicion that the breast lesion might be a granulomatous entity associated with erythema nodosum. Microbiological results from the abscess drainage indicated the presence of multisensitive Streptococcus epidermidis.

The patient was referred to internal medicine for follow-up. An extensive workup for infectious and autoimmune diseases was conducted, all of which returned negative results (Table 1).

**Table 1:** Infectious and rheumatologic workup.

Anti-HVI I, II	NR
HCV	NR
HBV	Not immune
Anti-*Treponema pallidum* antibody	Negative
*Borrelia burgdorferi* IgM and IgG	Negative
*Rickettsia conorii* IgM and IgG	Negative
Leptospira IgM	Negative
Leishmania spp. IgG	Negative
Interferon gamma release assay	Negative
Blood culture	Negative
Varicella-zoster virus IgG	Positive
ANA	Negative
p-ANCA (MPO)	Negative
c-ANCA (PR3)	Negative
Anti-CCP antibody	Negative
DsDNA antibody	Negative
C3 complement	1.73 g/L (0.83–1.93)
C4 complement	0.25 g/L (0.15–0.57)
Rheumatoid factor	Negative
Angiotensin-converting enzyme	<5 U/L (8–76)
IgG anti-PCP	55.4 μg/mL (>15.4 μg/mL)
IgG anti-PCP2	22.6 μg/mL (>5.4)
IgA	1.42 g/L (0.70–4.00)
IgM	1.53 g/L (0.40–2.30)
IgG	9.97 g/L (7.00–16.00)

One month after her hospitalization, the erythema nodosum had resolved spontaneously, while the granulomatous mastitis remained stable. Treatment options were discussed with the patient, who opted for conservative management with active surveillance and was started on prednisolone 30 mg/day.

**Fig. 3:**
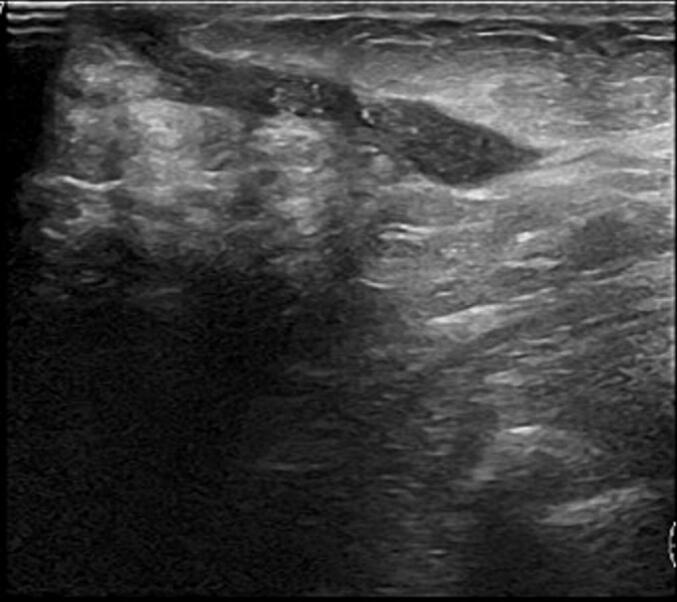
The ultrasound showed abscess coletion and a fistulous tract to the skin.

**Fig. 4:**
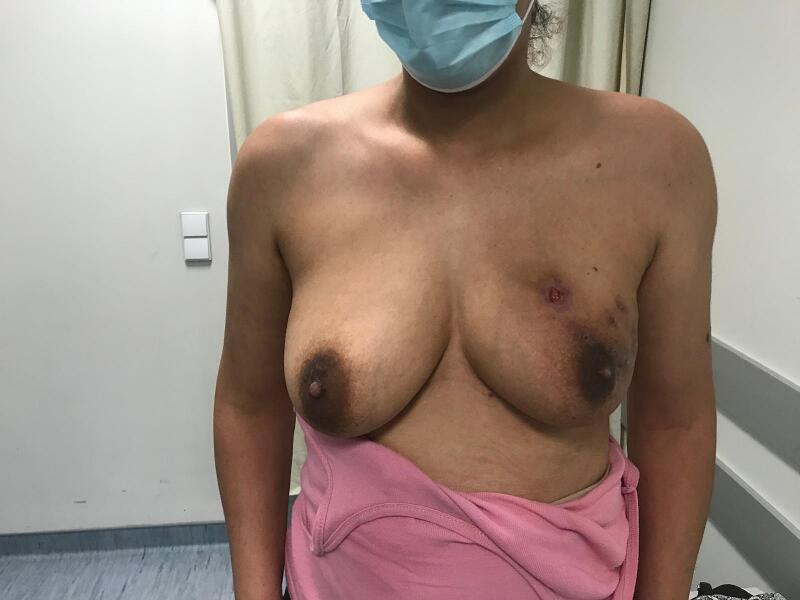
Right breast with multiple fistulous tracts to the skin.

**Fig. 5:**
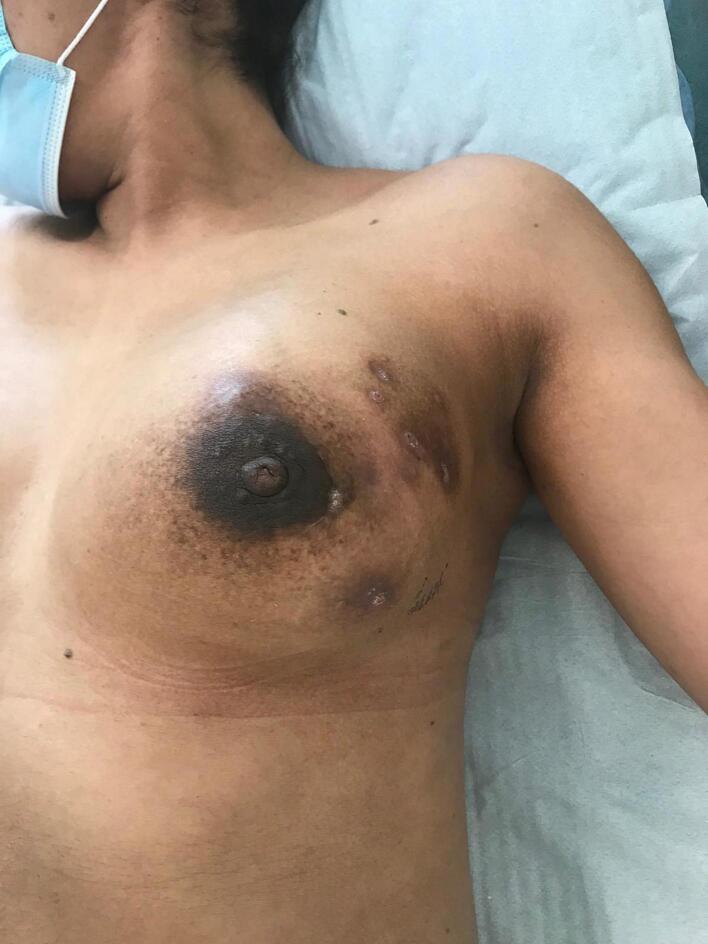
Right breast with multiple fistulous tracts to the skin.

**Fig. 6:**
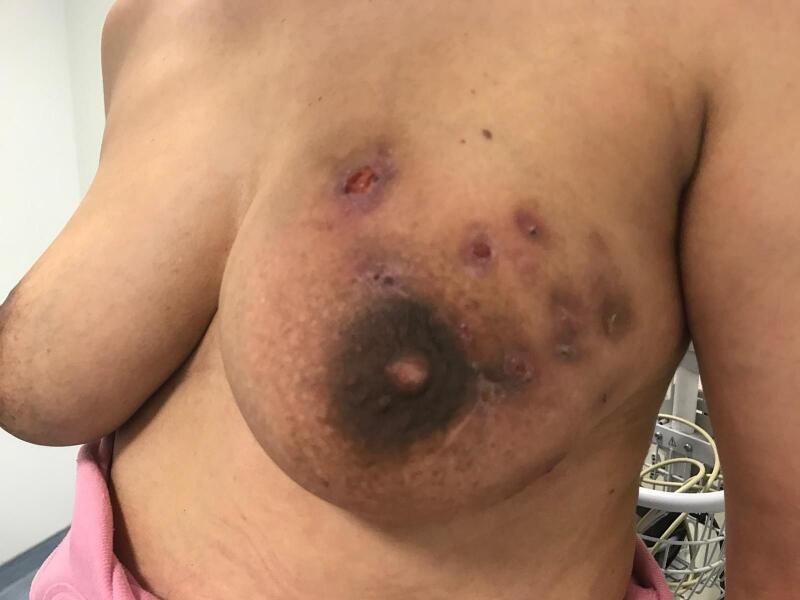
Right breast with multiple fistulous tracts to the skin.

Over a 1-year follow-up period she experienced several episodes of mastitis requiring percutaneous drainage and was prescribed multiple courses of antibiotics, including amoxicillin and clindamycin. Imaging studies consistently revealed multiple abscess collections and fistulous tracts to the skin. (Fig. 3, Fig. 4, Fig. 5, Fig. 6).

Due to the persistence of mastitis episodes and the fact that the patient was no longer breastfeeding, the initiation of treatment with methotrexate 5 mg once per week was proposed. Despite this treatment, the patient continued to experience episodes of mastitis, leading to an increase in the dose to 10 mg once per week.

Based on the clinical presentation and the workup performed, a differential diagnosis was established between sarcoidosis with breast involvement and idiopathic granulomatous mastitis. Consequently, a thoracoabdominopelvic CT scan was conducted, which revealed no mediastinal lymphadenopathy, cardiac or pulmonary abnormalities. Ophtalmologic examination was negative for uveitis and episcleritis. Based on the lack of these systemic findings, the diagnosis of idiopathic granulomatous mastitis with erythema nodosum was made.

With continued clinical improvement, particularly in breast-related findings, the patient remains on methotrexate therapy, which she has been tolerating well and responding favorably to.

## 3 Discussion

Idiopathic granulomatous mastitis is a chronic granulomatous inflammatory condition of the breast, characterized by non-necrotizing granulomas confined to the lobules, with no detectable infectious agents.

Despite ongoing research, its precise etiology remains unclear, and the lack of standardized diagnostic and therapeutic guidelines reflects the inconsistency in clinical understanding. Several potential contributing factors have been suggested, including infections, alpha-1 antitrypsin deficiency, hormonal influences such as oral contraceptive use, pregnancy, lactation, and hyperprolactinemia, as well as lifestyle and metabolic factors like smoking, trauma, diabetes, and autoimmune diseases [[7], [8], [9]].

As demonstrated in our case report, IGM predominantly affects women in their 30s and 40s and exhibits a strong correlation with a history of pregnancy and lactation.

The interplay between pregnancy, lactation, and autoimmune disease development has been extensively explored in medical literature. Both physiological states involve profound hormonal fluctuations, notably in estrogen, progesterone, and prolactin levels, which have significant immunomodulatory effects. These hormonal shifts may contribute to the initiation or aggravation of autoimmune disorders in genetically or immunologically predisposed individuals. Moreover, the mechanical and physiological stress associated with lactation, including microtrauma and the accumulation of milk proteins, may act as a stimulus for an autoimmune reaction within the breast interstitial tissue [[10], [11], [12], [13], [14], [15]].

T cell-mediated cytokine responses play a pivotal role in the pathogenesis and progression of autoimmune and inflammatory diseases. These immune mechanisms, while most active during pregnancy and the postpartum period, can exert long-term effects, potentially emerging years later. Pregnancy induces a predominantly T helper 2 (Th2)-oriented immune response due to hormonal regulation, which may exacerbate Th2-driven autoimmune conditions. Conversely, the suppression of Th1/Th17-mediated responses during gestation may lead to temporary remission in Th1/Th17-associated diseases. This intricate hormonal-immune interaction underscores the lasting impact of pregnancy on immune system dynamics and autoimmune disease susceptibility [15,16].

The breast microbiota, largely composed of commensal skin flora such as Propionibacteria, coagulase-negative Staphylococci, and Corynebacteria, can infiltrate mammary tissue through the ductal system. Structural alterations such as nipple retraction may predispose individuals to bacterial entry, potentially contributing to infection and inflammation. There are three prevailing hypotheses regarding the role of Corynebacterium in granulomatous lobular mastitis: it directly provokes an autoimmune response by breaching ductal integrity; it interacts with other immunogenic factors, such as retained milk, to elicit an inflammatory response; or it serves as a secondary pathogen that exacerbates an immune-mediated process initiated by other antigens [14].

The association between granulomatous mastitis and erythema nodosum (EN) was initially documented by Adams in 1987, who proposed a shared immunological basis for both conditions. Erythema nodosum is a septal panniculitis affecting blood vessels and characterized by the presence of painful, erythematous nodules, typically localized to the bilateral pretibial regions, though it may also manifest on the upper extremities [17]. Reported prevalence rates of EN among IGM patients vary significantly, ranging from 6.6 % to 58.1 % [[16], [17], [18], [19]].

Although the precise pathogenesis of EN remains poorly understood, it is hypothesized that heightened levels of circulating immune complexes, cytokines, and chemokines may contribute to its development in severe cases of IGM. These immunological factors may trigger septal panniculitis by facilitating neutrophil recruitment and activation in the subcutaneous tissue of the lower limbs [18]. The co-occurrence of systemic manifestations, including EN and arthritis, further supports the theory that IGM may have an underlying autoimmune component.

The simultaneous presentation of IGM and EN suggests a common immunopathogenic pathway, potentially driven by hypersensitivity or an autoimmune mechanism. Recognizing this link is essential for accurate diagnosis and optimal management, emphasizing the need for a multidisciplinary approach when evaluating patients with either condition [20,21].

This disease is recognized as a significant clinical mimic due to its resemblance to a wide range of conditions, including sarcoidosis, tuberculosis, malignancies, and infectious diseases. Proper differentiation of idiopathic granulomatous mastitis (IGM) from other autoimmune and granulomatous disorders is crucial for accurate diagnosis and management.

Bacterial infections that can lead to granulomatous inflammation include tuberculosis, leprosy, and cat-scratch disease, while fungal infections such as histoplasmosis, cryptococcosis, and coccidioidomycosis may also elicit a similar immune response [7,8]. Actinomycosis, which can cause chronic draining breast abscesses, is another infectious condition that may closely resemble IGM. Additionally, foreign body reactions to materials like silicone and beryllium, as well as fat necrosis, can present with overlapping clinical features [7].

Autoimmune-related granulomatous inflammation encompasses conditions such as Crohn's disease, sarcoidosis, and various forms of vasculitis, including granulomatosis with polyangiitis, giant cell arteritis, Takayasu's arteritis, and eosinophilic granulomatosis with polyangiitis (formerly Churg–Strauss syndrome) [7,9]. Lastly, inflammatory breast cancer must always be ruled out as part of a thorough diagnostic evaluation.

The management of IGM remains controversial, with no established gold standard. Treatment strategies range from close observation, immunosuppressive therapy, and surgical intervention, depending on disease severity and patient response.

IGM follows a self-limiting course in many cases, with spontaneous resolution reported in up to 50 % of patients within 6 to 24 months. Therefore, close monitoring without active intervention is a reasonable first-line approach for mild and localized cases, particularly after a confirmed diagnosis. Pain management with nonsteroidal anti-inflammatory drugs and patient reassurance are often sufficient. However, this strategy is only suitable after ruling out infectious and malignant causes through biopsy, imaging, and microbiological cultures [10,28].

For moderate to severe cases, medical treatment is the preferred approach, providing effective symptomatic control and often leading to durable resolution of inflammatory lesions.

Systemic corticosteroids have demonstrated efficacy in treating IGM, with most patients responding within weeks to months. Initial doses typically range from 30 to 40 mg of prednisone daily, followed by a gradual taper over 3–6 months to minimize recurrence risk. Prolonged steroid use, however, carries significant adverse effects, including hypertension, glucose intolerance, osteoporosis, and Cushing's syndrome. Therefore, corticosteroids should be used at the lowest effective dose for the shortest duration [22].

For patients with steroid-refractory disease or significant side effects, methotrexate (10–15 mg weekly) or azathioprine can be used as steroid-sparing agents. Methotrexate, commonly used in other granulomatous diseases such as sarcoidosis and giant cell arteritis, has shown remission rates of approximately 83 %, with a recurrence rate of 17 %. However, due to its teratogenicity, methotrexate is contraindicated in pregnant and breastfeeding women [23].

A recent study evaluating methotrexate monotherapy for IGM found that all patients initially achieved complete remission, although 17.6 % experienced relapse after tapering. However, these cases responded well to re-treatment with low-dose methotrexate. Overall, methotrexate appears to be a well-tolerated alternative, particularly for patients who cannot tolerate prolonged corticosteroid therapy [24].

Although IGM is a sterile inflammatory process, many patients initially receive empirical antibiotics due to the presence of erythema, induration, and abscess-like lesions, which can mimic bacterial mastitis. If an abscess is present, cultures should be obtained before initiating antibiotic therapy. Antibiotics should be discontinued if cultures are negative, as they do not alter disease progression in true IGM cases [25,26].

Given that hyperprolactinemia may contribute to IGM pathogenesis by overstimulating breast tissue, bromocriptine has been explored as an adjunctive therapy. A case series demonstrated a favorable response in 31 % of patients when combined with corticosteroids, suggesting that prolactin-lowering agents may have therapeutic potential in select cases [10].

Surgical excision is reserved for refractory cases or patients with persistent disease despite medical therapy. However, surgery is associated with a high recurrence rate, and wide excision may lead to cosmetic deformities and functional impairment. Therefore, it is generally not recommended as a first-line treatment unless complications such as abscess formation or fistulae necessitate intervention [27,28].

## 4 Conclusion

Idiopathic granulomatous mastitis is a diagnosis of exclusion and the clinical findings are non-specific.

This case highlights the importance of considering systemic conditions and concurrent medical events when interpreting breast images. Recent reports have noted an association between granulomatous mastitis and erythema nodosum, though the underlying pathophysiology of this link, as well as the optimal treatment and follow-up strategies, remain unclear. Further research is needed to better understand the relationship between these entities and to develop more effective management protocols.

## Registration of research studies

Not applicable.

## CRediT authorship contribution statement

Catarina Gil - writing the paper.

Alice Pimentel - writing the paper.

Rita Lages - writing the paper.

Ana Isabel Ferreira - writing the paper.

João Barros da Silva - writing the paper.

## Consent

Written informed consent was obtained from the patient for publication of this case report and accompanying images. A copy of the written consent is available for review by the Editor-in-Chief of this journal on request.

## Ethical approval

The study is exempt from ethical approval in our institution.

## Guarantor

- Catarina Gil
- Ana Isabel Ferreira

## Funding

No funding for research.

## Declaration of competing interest

No conflicts of interest.
